# Optical quantum super-resolution imaging and hypothesis testing

**DOI:** 10.1038/s41467-022-32977-8

**Published:** 2022-09-13

**Authors:** Ugo Zanforlin, Cosmo Lupo, Peter W. R. Connolly, Pieter Kok, Gerald S. Buller, Zixin Huang

**Affiliations:** 1 Scottish Universities Physics Alliance, Institute of Photonics and Quantum Sciences, School of Engineering and Physical Sciences, Heriot-Watt University, David Brewster Building, Edinburgh, EH14 4AS UK; 2 Dipartimento Interateneo di Fisica, Politecnico di Bari, 70126 Bari, Italy; 3 Department of Physics and Astronomy, The University of Sheffield, Hounsfield Road, S3 7RH Sheffield, UK; 4 Centre for Engineered Quantum Systems, Department of Physics and Astronomy, Macquarie University, Sydney, NSW Australia

**Keywords:** Quantum optics, Quantum metrology, Imaging and sensing

## Abstract

Estimating the angular separation between two incoherent thermal sources is a challenging task for direct imaging, especially at lengths within the diffraction limit. Moreover, detecting the presence of multiple sources of different brightness is an even more severe challenge. We experimentally demonstrate two tasks for super-resolution imaging based on hypothesis testing and quantum metrology techniques. We can significantly reduce the error probability for detecting a weak secondary source, even for small separations. We reduce the experimental complexity to a simple interferometer: we show (1) our set-up is optimal for the state discrimination task, and (2) if the two sources are equally bright, then this measurement can super-resolve their angular separation. Using a collection baseline of 5.3 mm, we resolve the angular separation of two sources placed 15 μm apart at a distance of 1.0 m with a 1.7% accuracy - an almost 3-orders-of-magnitude improvement over shot-noise limited direct imaging.

## Abstract (web summary)

Estimating the angular separation between two incoherent sources below the diffraction limit is challenging. Hypothesis testing and quantum state discrimination techniques are used to super-resolve sources of different brightness with a simple optical interferometer.

## Introduction

Hypothesis testing, parameter estimation, and imaging are fundamental scientific tasks that can all be improved using quantum techniques^1–3^. A judicious choice of quantum probe state or measurement observable can significantly improve the information gained in a measurement. These improvements can manifest in a multitude of ways. For example, the noise in an image may be reduced^4,5^, or the resolution of the image may be improved beyond the classical Rayleigh limit^6–9^. Other improvements include ghost imaging, where information is extracted from quantum light that has not directly interacted with the object^10,11^, and quantum-enhanced non-linear microscopy^12^. Quantum lithography^13,14^, and quantum sensing^15–17^ exploit entangled or correlated sources to enable precision beyond what is achievable classically. In microscopy, these techniques compete with classical super-resolution methods that use engineered sources that exhibit non-linear responses or exploit selective activation and bleaching of fluorophores^18–21^.

When source engineering is not an option, which is the case for astronomical observations, quantum techniques can beat the diffraction limit by unlocking all the information about amplitude and phase in the collected light. Traditionally, the resolution of an imaging system is limited by the the Rayleigh criterion^7^: the minimum angular separation that can be resolved is ${\theta }_{\min }\approx \lambda /D$, where *λ* is the wavelength and *D* is the diameter of the lens. A recent result for super-resolving a pair of incoherent sources has triggered much interest in the field^3^. It was shown that there is no loss of precision associated with estimating the sources’ angular separation, even when their separation is smaller than ${\theta }_{\min }$. However, prior to measuring the separation, one needs to ensure that there are two sources and not just one. One straightforward method would be to use direct imaging (DI) to determine whether a secondary source is present. In a diffraction limited system, the image of a point-like object is not a point but has a finite spread characterised by the point-spread function (PSF). If the two sources overlap on the image screen, this blurring presents a severe practical obstacle to direct detection of exoplanets^22,23^, especially when one source is much dimmer than the other.

**Fig. 1:**
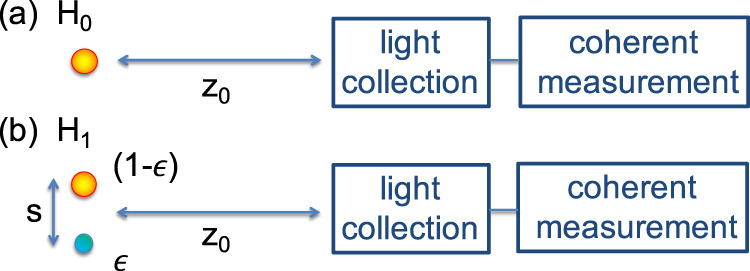
An optical imaging system is used to discern between two hypotheses, followed by parameter estimation. If hypothesis *H*_0_ is true (**a**), only one source of intensity *N* is present; if *H*_1_ is true (**b**), two sources are present, with total intensity *N* and the relative intensity is *ϵ*/(1 − *ϵ*). For *H*_1_, angular separation between the two sources is *θ* = *s*/*z*_0_.

Quantum hypothesis testing techniques, on the other hand, can be used (Fig. 1) when the task is to determine whether a secondary source exists^1^. The goal is to minimise the probability of a false negative (missing the second source). If we are happy to accept a certain probability of false positives (type-I error), then the probability of a false negative (type-II error) is given by the quantum Stein Lemma^24,25^. This asymmetric error setting is particularly applicable to rare events such as exoplanet identification^22,23^, or events with important ramifications such as dimer detection in microscopy^26^. In quantum information theory, the two hypotheses—one source versus two sources—are modelled by two quantum states, *ρ*_0_ and *ρ*_1_. We consider *n* detection events, and work in the regime of highly attenuated signals where *n* corresponds to the number of photons received. We define *α*_*n*_ and *β*_*n*_ as the probabilities of type-I and type-II errors, respectively. Given a bounded probability of a type-I error, *α*_*n*_ < *δ*, the quantum Stein lemma^27,28^ states that:

1 $${\beta }_{n}=\exp \left[-nD({\rho }_{0}|\vert {\rho }_{1})-\sqrt{nb}\,{{{\Phi }}}^{-1}(\delta )-O(\ln n)\right],$$

where

2 $$D({\rho }_{0}|\vert {\rho }_{1})={{{{{{{\rm{Tr}}}}}}}}\left[{\rho }_{0}(\ln {\rho }_{0}-\ln {\rho }_{1})\right],$$

is the quantum relative entropy (QRE)^29^, Φ(*δ*) is the Error Function, *b* is the variance of the QRE^30^ and ln indicates the natural logarithm. The quantum Stein Lemma and the quantum Cramér-Rao bound are usually defined in terms of number of copies of the state. However, here we work in the regime of highly attenuated signals and post-selected on photon detection events. Therefore, we refer to *n* as the number of photons detected. The two approaches differ only by a normalisation factor dictated by the overall transmission-detection efficiency. In the limit of large *n*, the leading term in the error exponent is the one proportional to the QRE *D*(*ρ*_0_∣∣*ρ*_1_). Therefore ${\beta }_{n}\simeq \exp \left[-nD({\rho }_{0}|\vert {\rho }_{1})\right]$ asymptotically. The quantum Stein lemma is already optimised over all possible measurements, therefore, it depends only on the two states to be discriminated, *ρ*_0_ and *ρ*_1_. The QRE provides a significant improvement in the error exponent *β*_*n*_ over the classical relative entropy for direct imaging^1^, thereby significantly reducing the probability of error, even when the two sources have small angular separations.

Once it is established with reasonable confidence that there are two sources, one can use quantum metrology to perform parameter estimation on the angular separation. The ultimate precision in the estimation is dictated by the quantum Cramér–Rao bound^31^. For any density matrix *ρ*(*θ*) with spectral decomposition $\rho (\theta )={\sum }_{i}{p}_{i}\left|{e}_{i}\right\rangle \left\langle {e}_{i}\right|$ that encodes the information of the parameter *θ*, the mean square error Δ^2^*θ* is lower bounded by the quantum Fisher information (QFI) *I*_*θ*_,

3 $${{{\Delta }}}^{2}\theta \ge \frac{1}{n{I}_{\theta }},\quad {I}_{\theta }=2\mathop{\sum}\limits_{i,j}\frac{\big\langle {e}_{i}|\,{\partial }_{\theta }\rho|{e}_{j}\big\rangle }{{p}_{i}+{p}_{j}},$$

where ∂_*θ *_*ρ* = ∂*ρ*/∂*θ*, and *n* is the number of photons detected where the summation is restricted to terms with $p_{i} + p_{j} \, > \, 0$. The QFI represents the ultimate precision limit for the estimation of the given parameter, which may be achieved by some particular measurement. Obviously, not all measurements allow us to achieve it. For any given measurement, which yields a particular distribution of measurement outputs, the optimal mean square error is bounded by its associated classical Fisher information (FI), which is the classical counterpart of the QFI. Here we describe a method, based on interferometry, to experimentally achieve the ultimate quantum Cramér–Rao bound^32^. If a lens is used and the PSF is approximately Gaussian, this ultimate bound can be achieved by spatial-mode demultiplexing (SPADE) or similar methods^3,33^. For estimating the transverse separation between two equally bright sources, the QFI has been shown to be finite and independent of the separation^3^. This is in contrast with DI, which allows us to estimate the separation with limited precision that drops to zero when the separation is small compared to the width of the PSF.

Sub-Rayleigh super-resolution imaging through coherent detection of incoherent light is currently an active area of research^5,34–47^. However, implementing the optimal measurement is typically non-trivial. In this paper we achieve two goals: (1) we experimentally demonstrate clear sub-Rayleigh scaling for quantum state discrimination of singular versus binary sources, and (2) we approach the quantum Cramér–Rao bound for estimating the angular separation of two sources with equal brightness. Most importantly, we significantly simplify the required experimental complexity. The two goals are achieved with a single measurement set-up: all the above tasks can be performed with a simple interferometer with two spatial modes, i.e., we collect photons at two spatial locations. Then, we perform photon counting at the output of the interferometer, and by analysing the statistics we can saturate both the QRE and the quantum Cramér–Rao bound.

## Results

### The Model

First, consider the task of discriminating between one source or two sources with a separation *s* in the object plane. Hypothesis *H*_0_ states that only one source is present, and it is positioned at *x*_0_. Hypothesis *H*_1_ states that two sources are present, where the first source is centred at *x*_0_, and it has an angular separation *θ* = *s*/*z*_0_ with the second. Furthermore, they have relative intensities (1 − *ϵ*) and *ϵ* respectively; without loss of generality, we assume *ϵ* ≤ 0.5. We will label a photon originating from the brighter source with intensity (1 − *ϵ*) as $\left|{\psi }_{{{{{{{{\rm{star}}}}}}}}}\right\rangle$, and the source with intensity *ϵ* as $\vert {\psi }_{{{{{{{{\rm{planet}}}}}}}}}\rangle$. The two states on the image plane are generally non-orthogonal. The density matrices associated with the two hypotheses *H*_0_ and *H*_1_ are, respectively

4 $${\rho }_{0}=\vert {\psi }_{{{{{{{{\rm{star}}}}}}}}}\rangle \langle {\psi }_{{{{{{{{\rm{star}}}}}}}}}\vert,$$

5 $${\rho }_{1}=(1-\epsilon )\vert {\psi }_{{{{{{{{\rm{star}}}}}}}}}\rangle \langle {\psi }_{{{{{{{{\rm{star}}}}}}}}}\vert+\epsilon|{\psi }_{{{{{{{{\rm{planet}}}}}}}}}\rangle \langle {\psi }_{{{{{{{{\rm{planet}}}}}}}}}\vert .$$

These two hypotheses can be discriminated by DI, in which case an optical system (which we may model as a thin converging lens) is used to create an image of the (unknown) source. The optical system is characterised by its PSF, which for a circular aperture is described by the Airy function. The latter, in turn, can be well-approximated by a Gaussian function with variance *σ*. In DI, the focused image is measured via pixel-by-pixel intensity detection, which in the weak-signal regime yields the empirical probability distribution of detecting a photon in each pixel. From the analysis of the data collected this way, one addresses the problem of hypothesis testing. The probability of a false negative is quantified by the classical analogue of the quantum Stein lemma, which expresses the error exponent in terms of the classical relative entropy (CRE), i.e., the Kullback–Leibler divergence. In the limit that *θ* ≤ *σ* and *ϵ* ≪ 1 the classical relative entropy from DI is approximately $(\exp ({\theta }^{2}/{\sigma }^{2})-1)\,{\epsilon }^{2}/2$^1^. This quadratic scaling in *ϵ* formally expresses the challenges of using DI for exoplanet detection, especially when the planet is much dimmer and very close to the star.

**Fig. 2:**
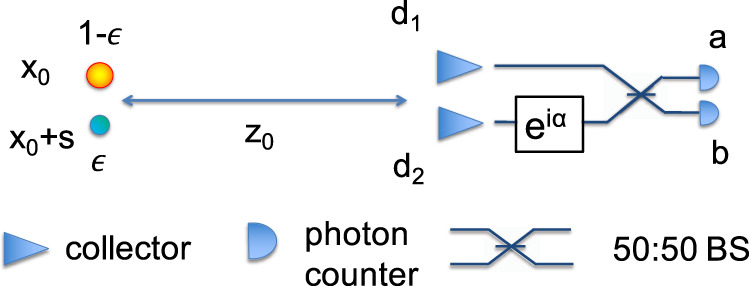
Schematic of two sources with a separation of *s* in the object plane, with relative intensities *ϵ* and 1 − *ϵ*, at a distance *z*_0_ from the collectors. Two collectors at *d*_1_ and *d*_2_ direct light into a two-input interferometer consisting of a phase shift of *α* and a 50:50 beam splitter, followed by photon counters. A collector can be any optical element that collects light. This could incorporate lenses or in the case of this experiment, two fibre connectors that present only the bare fibre core diameters in the direction of the source.

By contrast, the QRE provides a 1/*ϵ* improvement over the CRE^1^. An almost-optimal quantum measurement, SPADE^3^, is able to achieve linear scaling in *ϵ* by performing spatial Hermite–Gaussian mode sorting^1^. Though the SPADE device has recently been built and demonstrated^48,49^, the set-up is sensitive to misalignment of the sources’ centroid^3^, cross-talk^48^, and is unsuitable for large-baseline instrument devices—SPADE is suited for circular lenses and mirrors and building such optical components larger than 10’s of metres is infeasible. Here, we present an alternative approach with reduced experimental complexity that can also be adapted for large-baselines devices^50^. If instead of a lens we place two optical collectors, *d*_1_ and *d*_2_, separated by *d* = ∣*d*_1_ − *d*_2_∣, and at a distance *z*_0_ from the sources (see Fig. 2), then the states $\left|{\psi }_{{{{{{{{\rm{star}}}}}}}}}\right\rangle$ and $\left|{\psi }_{{{{{{{{\rm{planet}}}}}}}}}\right\rangle$ can be described as

6 $$\left|{\psi }_{{{{{{{{\rm{star}}}}}}}}}\right\rangle=\frac{1}{\sqrt{2}}\left(\left|{d}_{1}\right\rangle+{e}^{i\phi }\left|{d}_{2}\right\rangle \right),$$

7 $$\vert {\psi }_{{{{{{{{\rm{planet}}}}}}}}}\rangle=\frac{1}{\sqrt{2}}(|{d}_{1}\rangle+{e}^{i\psi }\vert {d}_{2}\rangle ),$$

where *ϕ*, *ψ* are the optical path differences of the sources to the two collectors. In the paraxial regime, these are

8 $$\phi \, \approx \, \frac{{{{{{k}}}}}\,d\,\theta }{2},\quad \psi\, \approx -\frac{{{{{{k}}}}}\,d\,\theta }{2},$$

where k is the wavenumber. Here we have assumed that the centre of the two collectors aligns with the centroid of the star-planet system for simplicity, but this is not necessary. In the limit of *ϵ* ≪ 1, the QRE between *ρ*_0_ and *ρ*_1_ is approximately (see Supplementary Information for details)

9 $$D({\rho }_{0}|\vert {\rho }_{1}) \, \approx \, \frac{{{{{{{k}}}}}}^{2}{\theta }^{2}{d}^{2}\epsilon }{4}.$$

Equation (9) is also linear in *ϵ*, thus has a factor 1/*ϵ* improvement compared to the classical counterpart; an optimal measurement that saturates the QRE (i.e., the measurement’s CRE that matches the QRE) is obtained by placing a phase shifter and a 50:50 BS after the two collectors, followed by photon counting. Given an imperfect interferometer with visibility *ν*, if there is no planet (*H*_0_ is true), then the probabilities that the photon is detected at detectors *a* or *b* are:

10 $${p}_{{H}_{0}}(a)=\frac{1}{2}\left[1+\nu \cos (\phi+\alpha )\right],$$

11 $${p}_{{H}_{0}}(b)=\frac{1}{2}\left[1-\nu \cos (\phi+\alpha )\right].$$

Where *α* is an adjustable phase. Otherwise, if *H*_1_ is true, then the probabilities are:

12 $${p}_{{H}_{1}}(a)=\frac{1}{2}[(1-\epsilon )(1+\nu \cos (\phi+\alpha ))+\epsilon (1+\nu \cos (\psi+\alpha ))],$$

13 $${p}_{{H}_{1}}(b)=\frac{1}{2}[(1-\epsilon )(1-\nu \cos (\phi+\alpha ))+\epsilon (1-\nu \cos (\psi+\alpha ))].$$

Given that we know the output probabilities of the two hypotheses, the CRE of this measurement is given by the classical version of Eq. (2), where

14 $$D({p}_{{H}_{0}}|\vert {p}_{{H}_{1}})=\mathop{\sum}\limits_{i\,\in \left\{a,b\right\}}{p}_{{H}_{0}}(i)\left[\ln {p}_{{H}_{0}}(i)-\ln {p}_{{H}_{1}}(i)\right].$$

The CRE is maximised for

15 $$\alpha \,\approx \, -{{{{{k}}}}}\,d\left[\epsilon ({x}_{0}+s)/{z}_{0}+(1-\epsilon )({x}_{0})/{z}_{0}\right],$$

and matches the QRE. Intuitively, this corresponds to the point where $|{p}_{{H}_{1}}(a)-{p}_{{H}_{0}}(a) \vert=\vert {p}_{{H}_{1}}(b)-{p}_{{H}_{0}}(b)|$ is maximised.

We now move onto performing quantum parameter estimation on the state. When the source intensities are equal, the QFI for the above state is ref. 32

16 $${I}_{\theta }=\frac{{{{{{{k}}}}}}^{2}{d}^{2}}{4},$$

which is constant in the effective pupil size *d* and independent of the angular separation *θ*. Hence, the angular separation can be estimated with constant precision even when its value is below the Rayleigh length, i.e., well beyond the diffraction limit. The very same measurement that achieves the maximum relative entropy, also allows us to saturate the quantum Cramér–Rao bound dictated by the QFI (i.e., the measurement achieves the minimum uncertainty).

Define ${a}_{{d}_{1}}^{{{{\dagger}}} ^{\prime} }({a}_{{d}_{2}}^{{{{\dagger}}} ^{\prime} })$ to be the creation operator at the collector position *d*_1_(*d*_2_). The adjustable phase shift *α* and the beam splitter transform the operator as

17 $${a}_{{d}_{1}}^{{{{\dagger}}} ^{\prime} }\to \frac{1}{\sqrt{2}}\left({a}_{{d}_{1}}^{{{{\dagger}}} }+{a}_{{d}_{2}}^{{{{\dagger}}} }\right),$$

18 $${a}_{{d}_{2}}^{{{{\dagger}}} ^{\prime} }\to \frac{{e}^{i\alpha }}{\sqrt{2}}\left({a}_{{d}_{1}}^{{{{\dagger}}} }-{a}_{{d}_{2}}^{{{{\dagger}}} }\right).$$

Applying the transformation in Eqs. (17) and (18) to the state $\rho=1/2\,(\vert {\psi }_{{{{{{{{\rm{star}}}}}}}}}\rangle \langle {\psi }_{{{{{{{{\rm{star}}}}}}}}} \vert+\vert {\psi }_{{{{{{{{\rm{planet}}}}}}}}}\rangle \langle {\psi }_{{{{{{{{\rm{planet}}}}}}}}}|)$, the probabilities of detecting the photon at either detector are

19 $${p}_{a}(\phi,\;\alpha,\;\nu )=\frac{1}{2}\left[1+\nu \cos (\alpha )\cos \left(\phi \right)\right],$$

20 $${p}_{b}(\phi,\;\alpha,\;\nu )=\frac{1}{2}\left[1-\nu \cos (\alpha )\cos \left(\phi \right)\right].$$

Here *ϕ* is the same as in Eq. (8). Determining *ϕ* statistically will provide an estimation on the angular separation, explained in the next sections. The maximum classical relative entropy and Fisher information are achieved around the phase values *α* = 0 or *π*. At these values, the CRE coincides with the QRE, and the Fisher information coincides with the QFI^32^.

### Experimental set-up

**Fig. 3:**
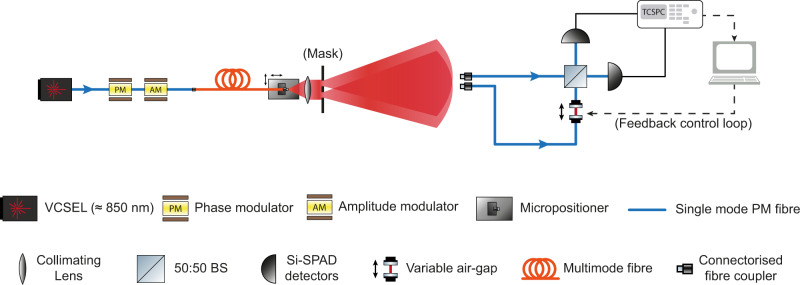
Experimental set-up. A VCSEL operated in pulsed mode generates coherent states that are phase and amplitude modulated to reproduce a pseudo thermal state. These states are then coupled into a multimode fibre and then collimated to a custom optical mask shaping the light beam into two pseudo-point-like sources. At 1 m distance, two single-mode polarisation-maintaining fibres collect the transmitted beam through connectorised couplers, followed by an adjustable air-gap that tunes the phase *α*, a 50:50 beam splitter and two single-photon detectors. These detectors are two Si-SPADs and register photon detection events storing their information onto a PC for post-processing via a TCSPC module. A feedback control system is used for interferometric stabilisation via the adjustable air gap.

The experimental set-up is depicted in Fig. 3. A fibre-coupled vertical cavity surface-emitting laser (VCSEL) with 848.2 nm central wavelength (0.11 nm FWHM) is operated in pulsed mode at a repetition rate of 1 MHz. This specific wavelength is chosen as it provides a good trade-off between single-photon detection efficiency (≈ 40%) with commercially available thick-junction silicon single photon avalanche diodes (Si-SPADs) detectors and tolerable optical loss in silica fibres (≈ 2.2 dB/km)^51^. The resulting coherent states are then coupled into two electro-optic modulators (EOMs) enabling phase and amplitude modulations of the individual coherent states. An external arbitrary waveform generator (AWG) electrically drives the two modulators by means of randomised modulation patterns so that the resulting optical states resemble a pseudo-thermal source^52^, required for the incoherent sources specified by the model and tested using a Hanbury Brown and Twiss interferometer. This modulation approach provides absolute control over each coherent state emitted by the source, including preserving the coherent state for use in interferometric measurements. In the results presented in this paper, we alternate the pseudo-thermal state with a coherent state that acts as a reference. The reference pulses provide the necessary interferometric stabilisation that is controlled via feedback from the two detectors, after the two pulses interfere at the beamsplitter. The alternate set of thermally modulated states instead are used to compute all relevant quantities detailed in our model (see Supplementary Information for further details).

After phase and amplitude modulation, the pseudo-thermal states are coupled into multimode optical fibres (8 m in length) in order to maximise mode dispersion and reduce wavefront spatial correlations due to the initial coupling of the VCSEL to single mode based optical components. The final thermal radiation is coupled into an adjustable aspheric collimator lens providing precise alignment with the remaining free-space optical components. Two pseudo-thermal sources are extracted from the collimated beam via a custom-made optical mask with two circular pinholes etched onto the surface, effectively reproducing two idealised point-like sources corresponding to the two distant stars of our model. Different etched patterns were fabricated using laser-written lithography in order to study a wide range of configurations with pinhole dimensions ranging from 10 to 50 μm in diameter and with spatial separation spanning from just 15 μm to almost 1 cm (see Supplementary Information for further details).

A neutral density filter is mounted on a separate movable micro-positioner block (not shown) placed in front of one of the two pinholes reducing the transmitted optical power through one of the pinholes. This configuration creates a controlled intensity imbalance between the two pseudo thermal sources effectively creating one bright source (a distant star) and one dimmer source (a distant exoplanet). At 1 m from the mask, two single-mode polarisation maintaining (PM) optical fibres, separated by 5.3 mm, are mounted on a micropositioner block (not shown) coupling the transmitted light beams into a balanced interferometer whose output modes are monitored by Si-SPAD detectors. The collectors used with the PM fibres are commercial fibre connectors with the bare fibre cores facing the approximate direction of the source. An adjustable air-gap is placed in one of the two optical paths allowing us to loss-balance the interferometer as well as providing direct control over the optical path-length difference. A time-correlated single photon counting (TCSPC) unit processes the generated timetags with 1 ps resolution enabling fast readout times as well as full digital post-processing. For each configuration of the set-up, 25 individual measurements are taken with a 5 s integration time in order to reduce Poissonian errors associated with photon-count data. An active feedback mechanism is implemented to ensure high interferometric visibility (> 99%) during the entire duration of the data acquisition by means of a piezoelectric actuator adjusting the path-length difference of the interferometer via the air-gap (see Fig. 3).

### Experimental results

**Fig. 4:**
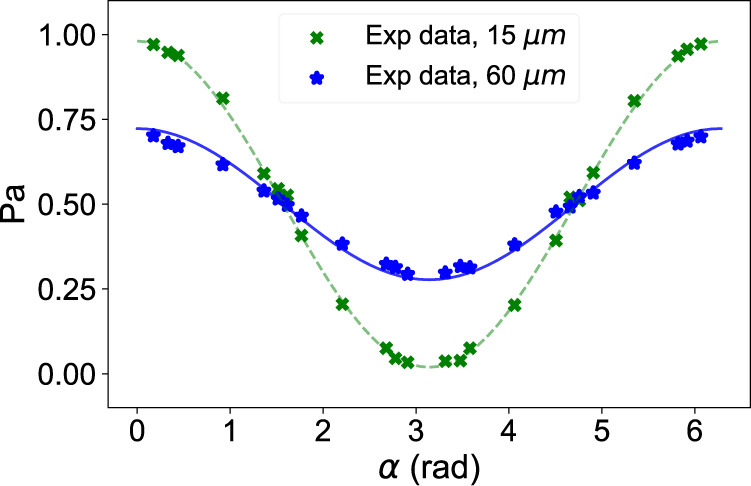
Photon detection probability of one of the detectors, as a function of the applied phase *α*, which is adjusted using the distance of the air-gap. The data shown are for *ϵ* = 0.5, for physical separations of 1.5 × 10^−5^ m and 6.0 × 10^−5^ m; the angular separations are 1.48 × 10^−5^ rad and 5.9 × 10^−5^ rad, respectively.

We experimentally measured the probability of the photon arriving at detectors *a* and *b*. As an example, in Fig. 4 we show the probability of the photon arriving at detector *a* for *ϵ* = 0.5 and angular separations of 1.48 × 10^−5^ rad and 5.9 × 10^−5^ rad. As expected, the contrast is higher for smaller separations: in the limit of small *θ*, the smaller the separation, the more spatially coherent the light becomes. In the limit that *θ* = 0, we have a point source and the visibility should be 100% in theory.

**Fig. 5:**
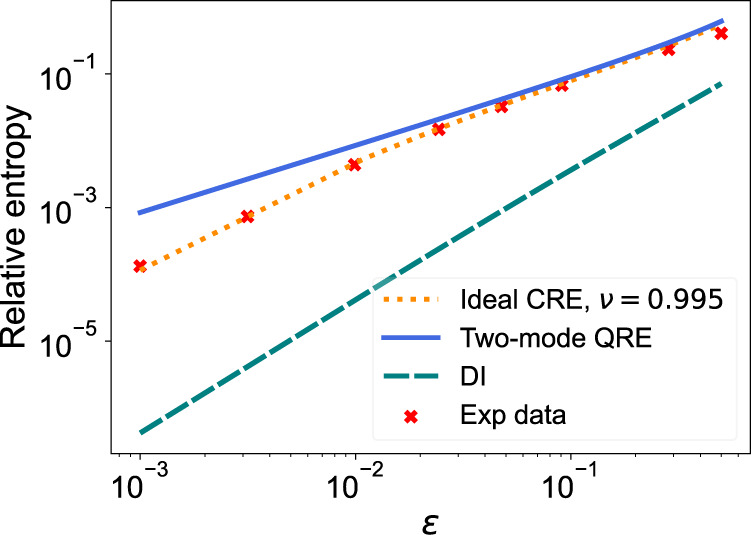
Relative entropy of the two hypotheses for different values of *ϵ*, using an angular separation of 5.9 × 10^−5^ rad. The plot shows: (1) the QRE of the two-mode state (blue solid line), (2) the CRE of the measurement maximised over *α*, given *ν* = 0.995 (orange dotted line), (3) the CRE for shot-noise limited direct imaging (DI, teal dashed line), and (4) the experimental data points (red crosses).

First, we compute the relative entropies of the two scenarios. In Fig. 5, we present the CRE of the measurement for different values of *ϵ* using an angular separation of 5.9 × 10^−5^ rad. For comparison, we also show the relative entropy for direct imaging using a lens with a diameter equal to the fibre separation of 5.3 mm (assuming a Gaussian PSF). Fig. 5 shows the distinct difference in scaling in *ϵ* between our method and DI. For *ϵ* > 10^−2^, we see that the two-mode CRE matches the two-mode QRE well. Due to experimental imperfections, around *ϵ* ~ 10^−3^ the achievable relative entropy has significantly deviated from the ideal quantum case, but still surpasses the DI limit by two orders of magnitude.

We now present the method of analysis and results for estimating the angular separation. We use maximum likelihood estimation to first extract the optical path difference *ϕ* between the source and the two collectors, and then obtain an estimator for the angular separation *θ*. Our method for extracting *ϕ* is a simpler version of the phase estimation method used in refs. 53, 54. We can determine *ϕ* directly from the detection statistics. To choose the estimator, it is useful to determine a probability density function for *ϕ* based on the detection results. The probability density function for *ϕ*, *P*(*ϕ*), can be determined from Bayes’ theorem as follows: prior to any detected photons, we assume no knowledge of *ϕ*, and the corresponding prior distribution is therefore *P*_0_(*ϕ*) = 1/(2*π*). After one detection event μ = *a*, *b* and adjustable phase *α*, we have

21 $$P(\phi|\mu,\;\alpha,\;\nu )\propto {{{{{{{\mathcal{P}}}}}}}}(\mu|\phi,\;\alpha,\;\nu )\,{P}_{0}(\phi|\alpha,\;\nu ).$$

where ${{{{{{{\mathcal{P}}}}}}}}(\mu \,|\,\phi,\;\alpha \;,\nu )$ is the update probability distribution. After *m* detection events, the vector of measurement outcomes is ${\overrightarrow{\mu }}_{m}=({\mu }_{1},\;{\mu }_{2},\ldots,\;{\mu }_{m})$, where each element μ_*j*_ ∈ {*a*, *b*}, with *j* ∈ [1, *m*], corresponds to the detector *a* or *b* that signalled the presence of the photon. The probability density function for *ϕ* is then^54^

22 $$P(\phi|{\overrightarrow{\mu }}_{m},\;\alpha,\;\nu )\propto {{{{{{{\mathcal{P}}}}}}}}({\mu }_{m}|\phi,\; \alpha,\; \nu )\,P(\phi|{\overrightarrow{\mu }}_{m-1},\;\alpha,\;\nu ),$$

where the proportionality constant is determined by normalising the distribution.

In order to obtain an analytic form for $P(\phi|{\overrightarrow{\mu }}_{m},\;\alpha,\;\nu )$, we express it as a Fourier series

23 $$P(\phi|{\overrightarrow{\mu }}_{m},\;\alpha,\;\nu )=\frac{1}{2\pi }\mathop{\sum }\limits_{k=-m}^{m}{a}_{k}{e}^{ik\phi },$$

where *a*_*k*_ depends on ${\overrightarrow{\mu }}_{m}$, *α* and *ν*. After each detection event, we can write the updated distribution in this Fourier form as well. For example, if detector *b* fires, then following from Eq. (20),

24 $${{{{{{{\mathcal{P}}}}}}}}(\mu=b|\phi,\;\alpha,\;\nu )=\frac{1}{2}\left[1-\nu \cos (\alpha )\cos (\phi )\right],$$

which we can rewrite as

25 $${{{{{{{\mathcal{P}}}}}}}}(\mu=b|\phi,\;\alpha,\;\nu )=\frac{1}{2}-\frac{1}{4}\,\nu \cos (\alpha )\,{e}^{i\phi } -\frac{1}{4}\,\nu \cos (\alpha )\,{e}^{-i\phi }.$$

Therefore the update coefficients are ${a}_{0}=\pi,\, {a}_{1}={a}_{-1}=-\frac{\pi }{2}\nu \cos (\alpha )$. The factor $\nu \cos (\alpha )$ is computed directly from the coherent state statistics (see Supplementary Information for detailed derivation). Before the first detection (the prior distribution), Eq. (23) contains only one term, *a*_0_ = 1. After each detection event the number of Fourier coefficients grows by 2 (the ± *m* terms in the Fourier expansion). The coefficients *a*_*k*_ are updated using Eqs. (19), (20) and (22).

**Fig. 6:**
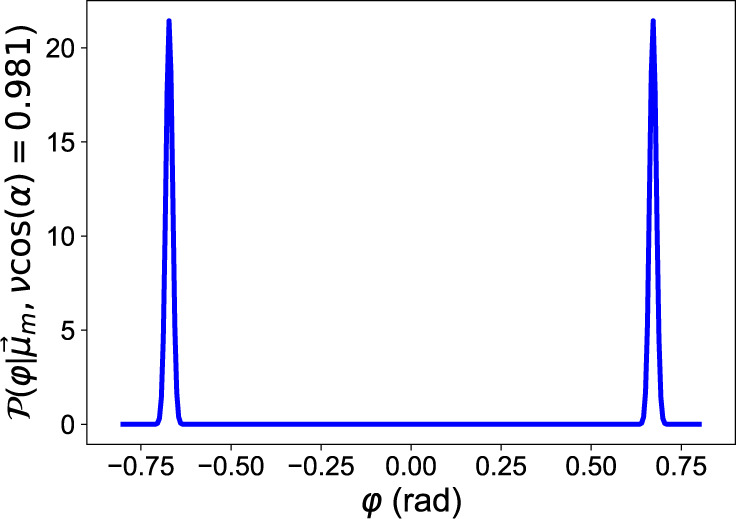
Simulated probability density function $P(\phi)$ with *ν* cos (α) = 0.981 . The probability density function for *ϕ*, after 12740 detection events of which 1478 were from detector *b*.

As an example, Fig. 6 shows the probability density function *P*(*ϕ*), calculated based on Eq. (23) after 12740 detection events where 1478 were output at detector *b*, with $\nu \cos (\alpha )=0.981$. Since $\cos (\phi )$ is an even function, there are two peaks, symmetrically placed around zero. We require only the magnitude of *ϕ* in the estimation of the angular separation *θ*.

Following maximum likelihood estimation, the value of *ϕ* at the maximum of *P*(*ϕ*) becomes our estimate, and the estimate of the separation *θ* is then given by

26 $${\hat{\theta }}_{{{{{{{{\rm{est}}}}}}}}}=2|\phi|/({{{{{k}}}}}d).$$

Once this estimate is obtained, we use the mean-square error (MSE) to quantify the precision, given by

27 $${{{{{{{\rm{MSE}}}}}}}}(\theta )={{{\Delta }}}^{2}\theta+{(\bar{\theta }-{\theta }_{{{{{{{{\rm{true}}}}}}}}})}^{2}.$$

Here $\bar{\theta }$ is the mean value of the estimates, and *θ*_true_ is the true value of the angle, which in this case is accessible via direct measurement. The MSE is equal to the variance for unbiased measurements and appropriately penalises biased estimates as well.

**Fig. 7:**
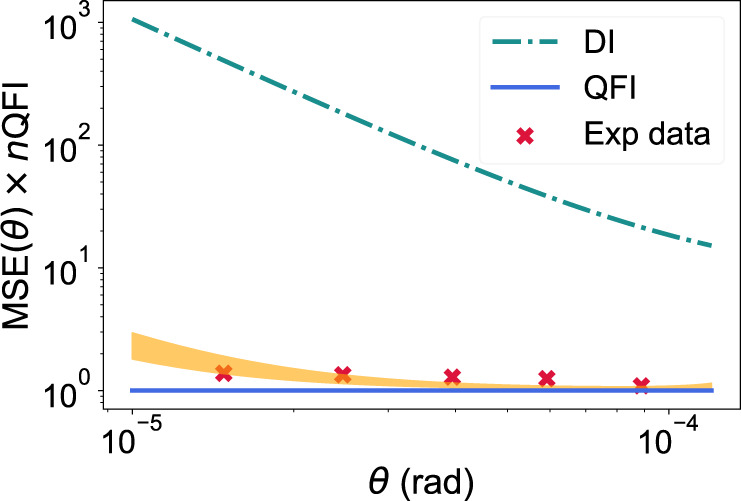
The mean squared error (MSE) of estimating the angular separation between two equally bright sources, normalised by the QFI, for different values of angular separation. The plot shows: (1) the normalised QFI, which is equal to 1 here, and is constant across the whole range of angular separations *θ* (blue solid line); (2) the MSE for shot-noise limited DI (teal dotted-dashed line); (3) the Fisher information for an interferometer with a visibility-phase factor $\nu \cos (\alpha )$ between 0.965 and 0.985 (orange shaded region); (4) the experimentally achieved MSE (red crosses).

For each value of the angular separation we obtained 25 different estimates, each detecting approximately *n* ≈ 60 000 photons. Figure 7 shows the MSE multiplied by *n* × *I*_*θ*_. The experimental data points are indicated by red crosses, and the achievable precision for shot-noise limited DI (using a circular lens of diameter 5.3 mm) is indicated by the dash-dotted line.

Experimentally, the data was collected with the factor $\nu \cos (\alpha )$ between 0.96 and 0.985. This is the shaded orange region in Fig. 7. The quantum Cramér-Rao bound is equal to 1 in this figure (blue solid line). We obtained unbiased estimates for values of angular separation *θ* that dramatically beat the Rayleigh limit. When *θ* = 1.48 × 10^−5^ rad, the root-mean-square errors are within 1.7% of the real value, which is two to three orders of magnitude more accurate than what is achievable with DI using a lens of the same diameter. For all measured angular separations, the MSE stayed within a factor 2 of the quantum Cramér-Rao bound.

## Discussion

In this work, we have analysed theoretically, and demonstrated experimentally, two tasks for super-resolution imaging based on hypothesis testing, quantum state discrimination and quantum parameter estimation. Estimating the angular separation between two sources is a challenging task for direct imaging, especially when their angular separation is smaller than the point spread function of the imaging system. The task of determining whether there are one or two sources is in itself a difficult task, especially when one source is much dimmer than the other.

We solved both these problems and, compared with previous works^1,33,55^, we have reduced the experimental complexity down to a simple two-input interferometer: we show that a simple set-up achieves sub-Rayleigh scaling for the state discrimination task, and if the two sources are of equal brightness, then this measurement can optimally estimate their angular separation, saturating the quantum Cramér–Rao bound.

We developed the theoretical analysis in the framework of single photons. However, the results extend to the regime of thermal sources as discussed in ref. 1. The experiment was conducted with weak thermal sources, where the probability of detecting multiple photons is highly suppressed. This reflects the fact that the measurement is post-selecting on single-photon events, which explains why the experimental data saturates the single-photon quantum limit. A similar observation was made for the problem of estimating the transverse separation^35^. In the absence of background noise, losses do not affect our resolution apart from reducing the total photon count.

Our experiment also shows a practical optical set-up that could potentially be integrated with current stellar interferometers. However, this would require a different approach for the phase stabilisation of the interferometer. For example, the stabilisation could be provided by a ground-based coherent source or an artificial guide star which are suitably multiplexed into the interferometry system.

Our set-up is compatible with existing two-mode interferometers: the two-mode model assumes that the dimension of the collectors is much smaller compared to the spatial separation of the collectors *d*. For optical interferometers where the separation between arms is of the order of ≈ 100’s m, collection using lenses and compound mirrors would be appropriate. For telescopes with point-spread functions that have 10’s of milliarcseconds in resolution, we expect our method to be able to distinguish, or measure the separation of binary stars to precisions well-above direct imaging. As an example, the exoplanet LkCa 15 c^56^ was observed with a Large Binocular Telescope (LBT) with a diffraction-limited PSF of ≈29 milliarcseconds (wavelength at 2.18 μm, 7 m in baseline). Using our method, if the instrument has visibility *ν* ≥ 98% (achieved by CHARA^57^), such an instrument can resolve two binary stars with separations less than 10 milliarcseconds (see Supplementary Information).

In order to achieve the desired precision, we need a sufficient number of photon counts acquired over a time period during which the phase is stabilised. Naturally, the phase stabilisation will be highly dependent on the environment. One approach is that our system could be entirely translated into a planar waveguide architecture where light from a telescope could be collimated directly into laser-inscribed couplers greatly reducing optical losses and improving phase stabilisation due to the reduced dimensions of the interferometer^58^. Moreover, our system could improve the MSE of estimating even smaller angular separations by increasing the number of collected photons, but higher interferometric visibility levels (≈ 99%) would be necessary to avoid signal degradation due to sub-optimal *α* values.

In our work we used pulsed light for both the reference signals and the pseudo thermal states. In a practical implementation, a celestial body would show a continuous form of radiation with a broad optical spectrum^59,60^ which limits the interferometric visibility. However, our system can be easily adapted to implement narrow bandpass filters to select the right bandwidth for the detection stage at the cost of a reduced photon level. Moreover, Si-SPADs could be replaced with SNSPDs for faster sampling time, higher detection efficiencies and reduced dark counts and timing jitter.

Recently, there has been a renewed interest in two-photon interferometry (intensity interferometry)^61,62^. Compared to those techniques, our method requires phase-stabilisation of the interferometer, but makes use of every photon received. The two-photon methods can achieve a very large baseline without needing an optical link between the system (or phase stabilisation), but suffer from low probability of successful detection events. In principle, if one has access to a quantum-enabled large baseline optical interferometer of the same baseline (such as those described in ref. ^63^), our scheme achieves much higher precision.

Here, we have focused on the most simple scenario of discriminating one versus two point-like sources, using a two-mode interferometer. Future work could explore the hypothesis testing for discriminating between multiple sources of different brightness, composite hypothesis testing, and the number of modes the interferometer would require for such tasks.

## Methods

### Pseudo thermal source generation

Thermal radiation is a semi-classical form of radiation characterised by a well defined optical intensity but undefined phase^64^. Its representation on a phasor diagram is that of a symmetric blurred circle centred around the axes’ origin (see Supplementary Fig. 1). Thermal states are generally associated with optical radiation with a reduced temporal and spatial coherence^65,66^ which limit their use for interferometric measurements. However, our work required precise control over the interferometer’s reference phase which could not be achieved by solely implementing a thermal source like an LED. Thankfully, the semi-classical nature of thermal states allows us to express their mathematical representation as a collection of individual coherent states weighted by a suitable quasi-probability distribution, i.e., the Glauber–Sudarshan *P*-function^67^:

28 $${\rho }_{{{{{{{{\rm{th}}}}}}}}}=\int P(\alpha )\left|\alpha \right\rangle \left\langle \alpha \right|{{{{{{{{\rm{d}}}}}}}}}^{2}\alpha,$$

where *P*(*α*) is the normalised *P*-function.

Referencing Fig. 3, the phase and amplitude modulations required to reproduce the correct P-function of a thermal state were provided via the two electro-optic modulators (EOMs) fibre-coupled to the laser source, a vertical cavity surface-emitting laser (VCSEL). A dual-channel arbitrary waveform generator provided independent electrical driving voltages to the EOMs which in turn applied a variable phase and amplitude modulation onto the individual coherent states generated by the VCSEL. Both modulators were LiNbO_3_ based with low insertion and coupling loss (≈ 0.25 dB) and low DC control voltages (≈ 2.5 V for phase inversion). The amplitude modulators comprised two laser inscribed waveguides configured in a Mach–Zehnder pattern where an external RF signal would change locally the refractive index of one arm actively altering the output power of the device. In order to reduce any external interference during their operation, the EOMs were mechanically and thermally isolated from the environment enhancing their operational stability. The final pseudo thermal source was tested by means of a Hanbury Brown and Twiss like experiment^68^ where second order correlations between the two detectors monitoring the interferometer were measured for different time delays via the QuCoa analysis software for the HydraHarp 400 (PicoQuant) TCSPC module. Supplementary Fig. 2 shows the computed *g*^(2)^(*τ*) as a function of the time delay between the two detectors for a pulsed source with a repetition rate of 1 MHz. The results showed a maximum at zero delay of *g*^(2)^(0) = 1.977 ± 0.003, in close agreement with the theoretical value of a true thermal source.

### Interferometric calibration

The experimental set-up depicted in Fig. 3 relied on spatial mode sorting of the pseudo thermal states via interferometric means. However, precise and reliable control over the optical path difference of the device was paramount for the desired sorting operation. Therefore, we implemented an active feedback loop mechanism to have direct control over the interferometer and the relative phase difference of its inputs. A reference coherent signal was multiplexed into the input signals via the same EOMs used for the generation of the pseudo thermal states. This mechanism effectively halved the final repetition rate to 500 KHz since every two laser pulses, one was used for calibration and tuning operations. Supplementary Fig. 3 depicts a simplified representation of a one-shot modulation signal used for the phase changing EOM. The first signal applies a voltage that imprints a complete *π* shift onto the coherent state while the second signal applies a random phase uniformly extracted from the set [0, 2*π*) ensuring that the final P-function was not skewed due to limited randomness generation^69,70^. The adjustable air-gap placed in one of the optical path of the interferometer was then used to ensure that the phase applied to the reference signal state was kept constant throughout the detectors’ integration time thus resulting in high interferometric visibility (≈ 99 %).

## Supplementary information

Supplementary Information

## Data Availability

All data generated in this study have been deposited in the Heriot-Watt University database^71^.
