# Supplementary material for: Optical quantum super-resolution imaging and hypothesis testing
Source: Nat Commun. 2022 Sep 13;13:5373. doi: 10.1038/s41467-022-32977-8 (PMC9470588; doi:10.1038/s41467-022-32977-8)
Supplement: Supplementary file 1 — Supplementary Information [file 41467_2022_32977_MOESM1_ESM.pdf]

Supplementary Information for "Optical quantum super-resolution imaging and hypothesis testing"

Ugo Zanforlin,[1,*] Cosmo Lupo,[2] Peter W. R. Connolly,[1] Pieter Kok,[3] Gerald S. Buller,[1] and Zixin Huang[3,4,†]

[1] *Scottish Universities Physics Alliance, Institute of Photonics and Quantum Sciences, School of Engineering and Physical Sciences, Heriot-Watt University, David Brewster Building, Edinburgh EH14 4AS, United Kingdom*

[2] *Dipartimento Interateneo di Fisica, Politecnico di Bari, 70126 Bari, Italy*

[3] *Department of Physics and Astronomy, The University of Sheffield, Hounsfield Road, S3 7RH Sheffield, United Kingdom*

[4] *Center for Engineered Quantum Systems, Department of Physics and Astronomy, Macquarie University, Sydney, Australia*

(Dated: August 6, 2022)

I. PSEUDO THERMAL SOURCE GENERATION

This section contains the representation of a thermal state in the phase space together with the experimental evaluation of the second order autocorrelation function $g^2(\tau)$ for said state. See subsection A of the Method section in the main manuscript for a more detailed description and analysis.

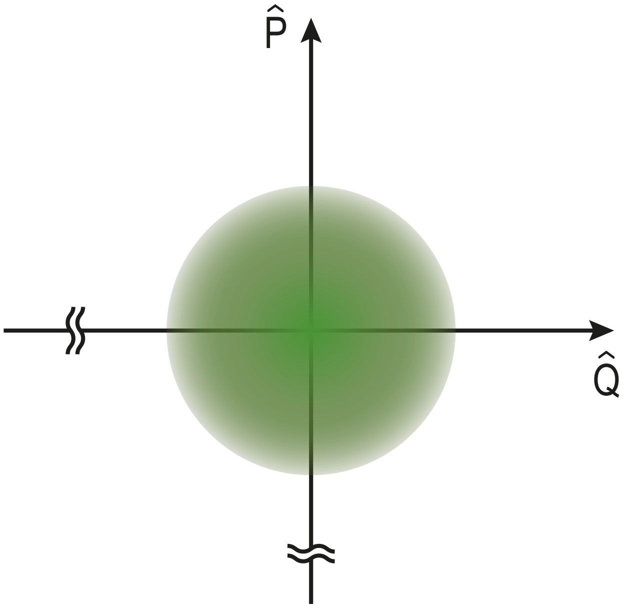

SUPPLEMENTARY FIG. 1. Phasor diagram representation of a thermal state. This state has a well defined mean photon number but undefined phase. $\widehat{\mathrm{P}}$ and $\widehat{\mathrm{Q}}$ are the momentum and position operators respectively associated with a quantum harmonic oscillator framework.

* U.Zanforlin@hw.ac.uk

† zixin.huang@mq.edu.au

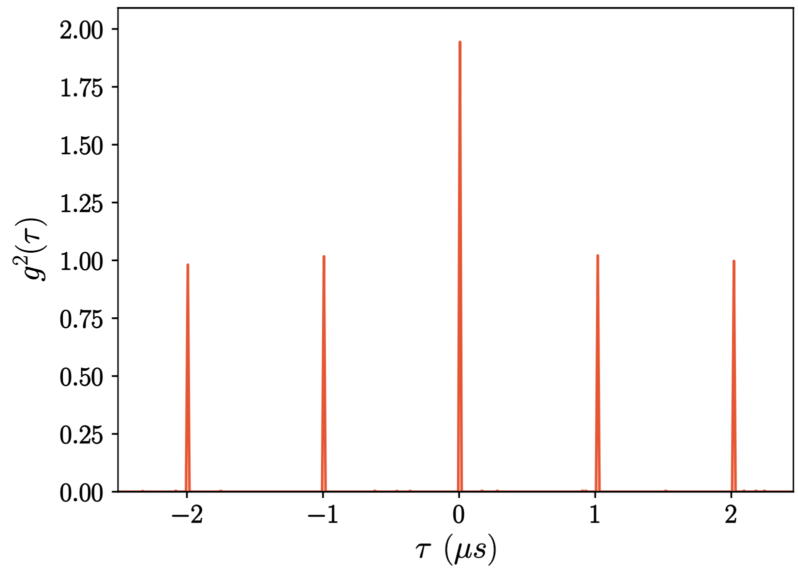

SUPPLEMENTARY FIG. 2. Experimental $g^{(2)}(\tau)$ autocorrelation function of the pseudo thermal source. The central peak at zero delay time shows a $g^{(2)}(0) = 1.977 \pm 0.003$ which is in close agreement with a theoretical value of a true thermal state. The coincidence peaks are separated by 1 μs which is consistent with the clock repetition rate of 1 MHz. All results have been collected in real-time via the QuCoa (PicoQuant) software with a total integration time of 60 s in order to limit evaluation errors.

II. INTERFEROMETRIC CALIBRATION

This section shows the diagram of a one-shot phase modulation electrical signal used to generate a thermal state via an electro-optic modulator (EOM). See subsection B of the Method section in the main manuscript for a more detailed description.

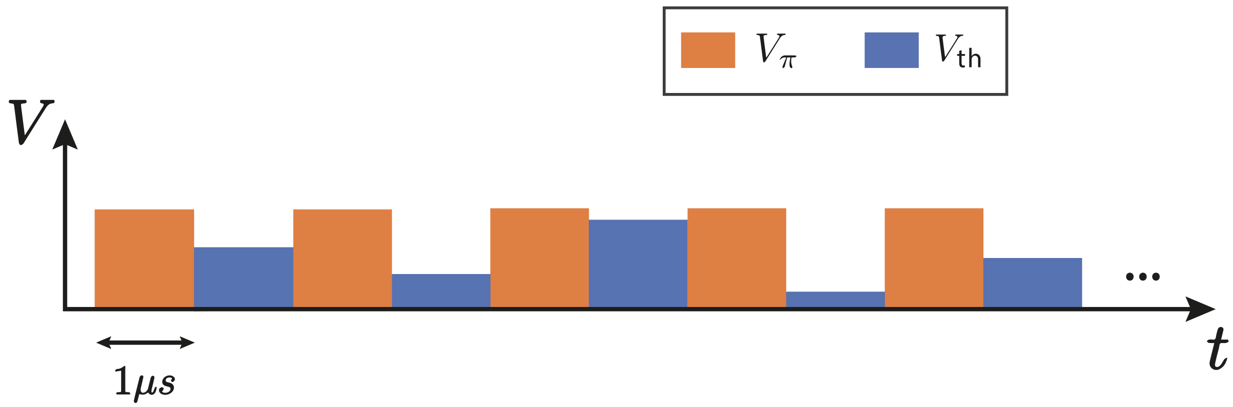

SUPPLEMENTARY FIG. 3. Diagram of a one-shot phase modulation signal to the EOM. The multiplexed reference signal is repeated every two electrical pulses (orange rectangles) effectively halving the clock repetition rate to 500 KHz. The thermal signal instead (blue rectangles) is selected uniformly at random within the range $[0, 2\pi)$ and similarly repeated every other electrical signal. The duration of all pulses is matched to the clock repetition rate of 1 MHz, i.e. 1 μs and the final pattern is then repeated every 200 ms.

III. OPTICAL MASK FABRICATION AND CHARACTERISATION

The optical masks were formed using patterned etching of thin chromium layers on a fused silica substrate. The 1.5 mm thick fused silica substrates were coated with 90 nm thickness of chromium using an electron-beam vacuum evaporation process forming a layer sufficiently thick to be fully opaque to the near-infrared radiation used in this experiment. The substrate was then coated in 5 nm positive photoresist (AZ 1505), and the pattern (pinholes, reference and alignment markers) inscribed using a Heidelberg DW66+ laser-writer. Once developed, the chromium was removed using a chemical etchant (TechniEtch Cr01). Fig. 4 displays pictures of the masks taken with a Leica DMRM microscope (15x magnification).

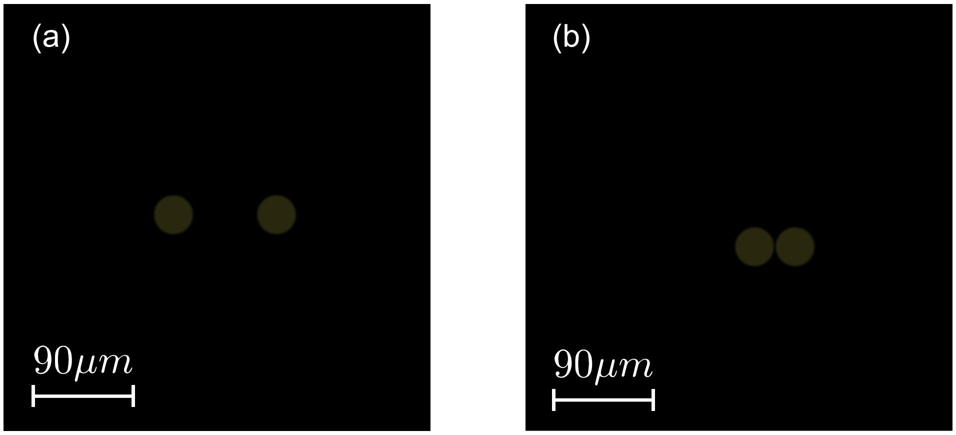

SUPPLEMENTARY FIG. 4. Pictures of the final optical masks taken with a Leica DMRM microscope (15x magnification). Image (a) shows a mask with 30 μm wide pinholes separated by 90 μm while image (b) shows a mask with pinholes of the same size but a 35 μm separation. Pictures' contrast has been saturated to better resolve the two pinholes against the background.

A single-photon sensitive CCD camera (Rolera EM-C^2 Bio-Imaging Microscopy Camera) was used to extract profile intensity images of the transmitted light by the two circular pinholes at different distances. Fig. 5 shows the normalised intensities at imaging distances $z = 2, 3$ and 20 cm for a mask with 30 μm wide pinholes separated by 1 mm using thermal radiation. At short distances, the intensity profiles depict the classical Airy diffraction pattern expected from circular apertures where faint secondary rings are visible. In these configurations, the two sources can still be resolved, however, as the distance between the masks and the camera increases, diffraction prevails and the profiles merge together removing any knowledge of the initial sources.

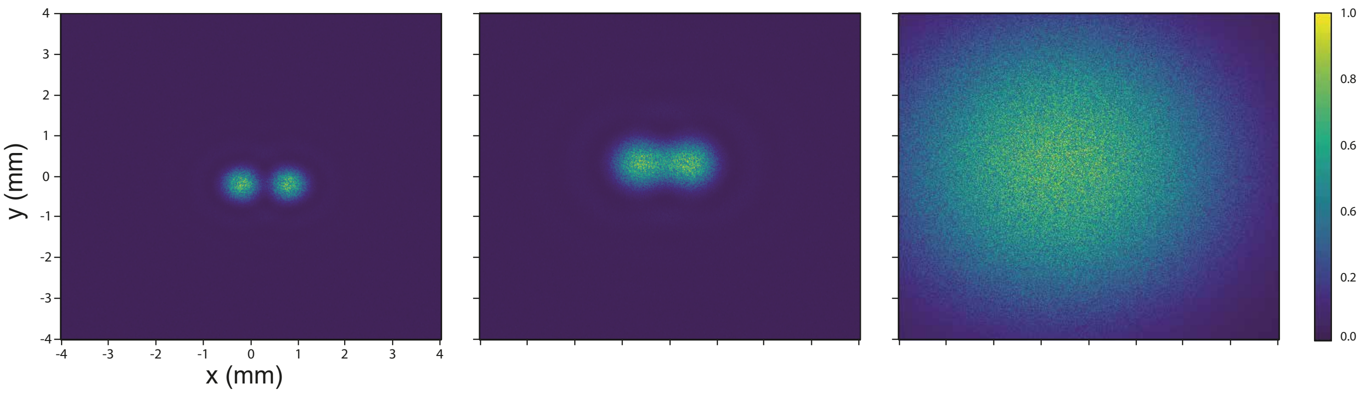

SUPPLEMENTARY FIG. 5. Normalised intensity profile pictures of transmitted light through the optical mask. At close imaging distances (a) $z = 2$ cm the two pseudo thermal sources are clearly distinct depicting two Airy diffraction patterns with faint secondary rings. As the distance increases, (b) $z = 3$ cm the two images merge together until they completely coalesce (c) $z = 20$ cm resulting in a heavily diffracted image where it is impossible to distinguish the individual sources. All pictures are relative to the same mask with 30 μm wide pinholes separated by 1 mm.

The masks were also tested using coherent radiation to ensure that the thermal generation process successfully removed any spatial correlation that could potentially disrupt the interferometer's mode sorting mechanism. Fig. 6 shows the resulting image for a mask with 30 μm wide pinholes separated by 150 μm placed at a distance $z = 15$ cm from the camera. Interferometric fringes are clearly recognisable showing a fringe separation of ≈ 0.821 mm which is in good agreement with the theoretical value expected for this imaging system.

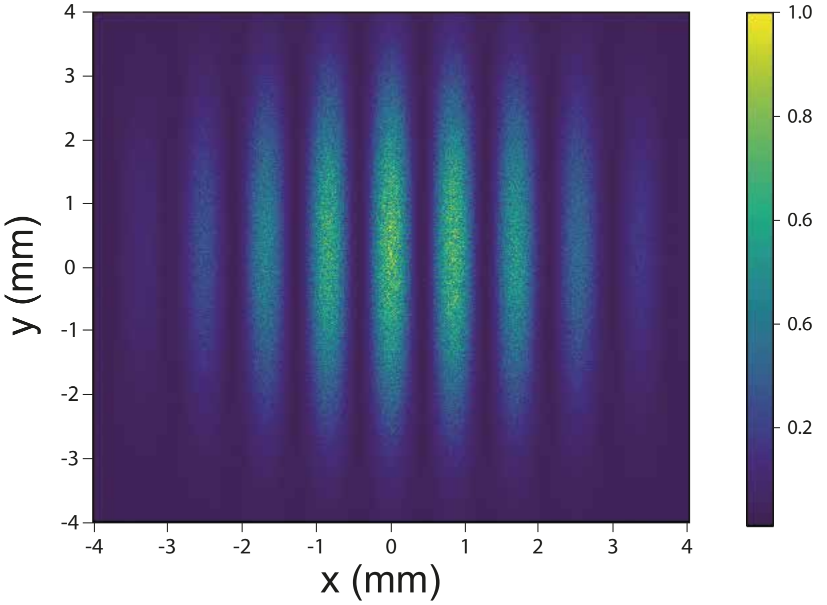

SUPPLEMENTARY FIG. 6. Normalised intensity profile picture of transmitted coherent light through the optical mask. Without any phase and intensity modulation provided by the EOMs, the two point-like sources undergo "classical" interference as demonstrated by the visible interferometric fringes. The mask used had 30 μm wide pinholes separated by 150 μm placed at a distance of 15 cm from the camera. The separation of the fringes is ≈ 0.821 mm which is consistent with the expected theoretical value for the imaging system used.

IV. QUANTUM STATE DISCRIMINATION

In a previous work [1], we have shown that a two-mode interferometer has the same sensitivity in estimating the separation between two sources as SPADE, given comparable numerical apertures. Here we show that the same two-mode interferometer is also optimal in our discrimination problem.

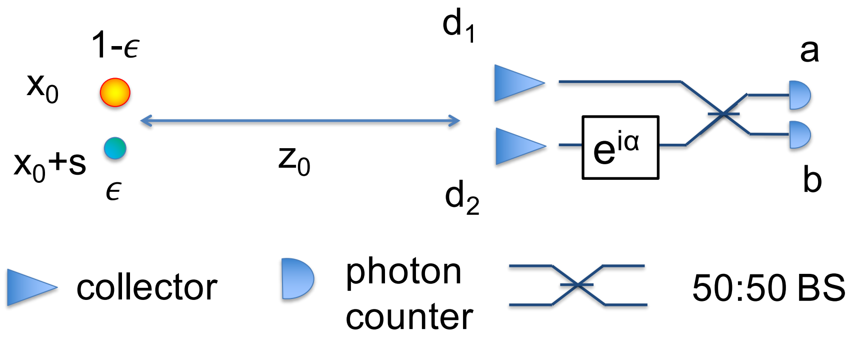

SUPPLEMENTARY FIG. 7. Schematic of two sources with a separation of s in the object plane, at a distance z_0 from the collectors. Two collectors at d_1 and d_2 direct light into a two-mode interferometer consisting of a phase shift of α and a 50:50 beam splitter, followed by photon counters.

Consider the set-up in Fig. 7 where two collectors are placed at positions d_1 and d_2 orthogonal to the optic axis; the collectors are at a distance z_0 from the sources. One source (the star) is positioned at x_0, and the planet, if present, is positioned at $x_0 + s$.

Assuming we are in the paraxial regime, the optical path difference of the planet between the two collectors is

$$\psi \approx \mathbf{k} z_0 \left(\frac{(x_0 + s - d_2)^2}{2z_0^2} - \frac{(x_0 + s - d_1)^2}{2z_0^2} \right)$$

$$= \mathbf{k} \left(\frac{d_2^2 - d_1^2}{2z_0} + \frac{(d_1 - d_2)(x_0 + s)}{z_0} \right) , \quad (1)$$

$$|\psi_{\text{planet}}\rangle = \frac{1}{\sqrt{2}} \Big(|d_1'\rangle + e^{i\psi} |d_2'\rangle \Big) . \quad (2)$$

The optical path difference of the star to the two collectors is

$$\phi \approx \mathbf{k} \left(\frac{d_2^2 - d_1^2}{2z_0} + \frac{(d_1 - d_2)x_0}{z_0} \right) , \quad (3)$$

$$|\psi_{\text{star}}\rangle = \frac{1}{\sqrt{2}} \Big(|d_1'\rangle + e^{i\phi} |d_2'\rangle \Big) . \quad (4)$$

where $\mathbf{k}$ is the wavenumber of the radiation. The states to discriminate between are:

$$\rho_0 = |\psi_{\text{star}}\rangle\langle\psi_{\text{star}}| , \quad (5)$$

$$\rho_1 = (1 - \epsilon) |\psi_{\text{star}}\rangle\langle\psi_{\text{star}}| + \epsilon |\psi_{\text{planet}}\rangle\langle\psi_{\text{planet}}| . \quad (6)$$

We define the angular separation $\theta = s/z_0$. In the basis of ρ_0, the two density matrices are

$$\rho_0' = \begin{pmatrix} 1 & 0 \\ 0 & 0 \end{pmatrix} , \quad (7)$$

$$\rho_1' = \begin{pmatrix} \frac{1}{2}(\epsilon \cos(\mathbf{k}\theta d) - \epsilon + 2) & \frac{1}{2} i\epsilon \sin(\mathbf{k}\theta d) \\ -\frac{1}{2} i\epsilon \sin(\mathbf{k}\theta d) & \epsilon \sin^2\left(\frac{1}{2}\mathbf{k}\theta d\right) \end{pmatrix} , \quad (8)$$

$$d \equiv d_1 - d_2 . \quad (9)$$

The QRE between ρ_0 and ρ_1 is approximately

$$D(\rho_0||\rho_1) \approx \frac{\mathbf{k}^2\theta^2 d^2\epsilon}{4}\,. \tag{10}$$

Now, we apply the measurement in Fig. 7. We put the collected light at d_1, d_2 through a phase shift $e^{i\alpha}$, followed by a 50:50 BS. We assume the operators transform as

$$a_{d1}^{\dagger'} \to \frac{1}{\sqrt{2}}\left(a_{d1}^{\dagger} + a_{d2}^{\dagger}\right), \tag{11}$$

$$a_{d2}^{\dagger'} \to \frac{e^{i\alpha}}{\sqrt{2}}\left(a_{d1}^{\dagger} - a_{d2}^{\dagger}\right). \tag{12}$$

For H_0, the measurement outcomes are

$$p_{H_0}(a) = \frac{1}{2}\Big[1+\cos(\phi+\alpha)\Big]\,, \tag{13}$$

$$p_{H_0}(b) = \frac{1}{2}\Big[1-\cos(\phi+\alpha)\Big]\,. \tag{14}$$

For H_1, they are

$$p_{H_1}(a) = \frac{1}{2}(1-\epsilon)\Big[1+\cos(\phi+\alpha)\Big] + \frac{1}{2}\,\epsilon\Big[1+\cos(\psi+\alpha)\Big]\,, \tag{15}$$

$$p_{H_1}(b) = \frac{1}{2}(1-\epsilon)\Big[1-\cos(\phi+\alpha)\Big] + \frac{1}{2}\,\epsilon\Big[1-\cos(\psi+\alpha)\Big]\,. \tag{16}$$

Define

$$\chi \equiv \frac{d_2^2 - d_1^2}{2z_0^2}\,, \tag{17}$$

we set α to

$$\alpha = -\chi - \left[\frac{\mathbf{k}\epsilon d\,(x_0+s)}{z_0} + \frac{(1-\epsilon)(\mathbf{k}x_0 d)}{z_0}\right]. \tag{18}$$

This is analogous to the SPADE method, where we align the apparatus to the weighted center. That is, the optical system will be aligned towards the weighted center (centroid). If only the star is present, then the centroid corresponds to the position of the star. Otherwise, it is somewhere between the star and the planet, namely, $x_0(1-\epsilon)+(x_0+s)\epsilon$. As an example, we could observe a blurred image without being able to discriminate the two point-sources, yet we can still identify the point of maximum apparent intensity and point the telescope in its direction. In other words, we are assuming a preliminary phase in which the position of the centroid has been estimated.

The classical relative entropy of this measurement is

$$\begin{aligned} D(p_0||p_1) = &\cos^2\left(\frac{1}{2}\mathbf{k}d\theta\epsilon\right)\left[\ln\left(\cos^2\left(\frac{1}{2}\mathbf{k}d\theta\epsilon\right)\right) - \ln\left(\frac{1}{2}(\epsilon\cos(\mathbf{k}d\theta(\epsilon-1)) - (\epsilon-1)\cos(\mathbf{k}d\theta\epsilon)+1)\right)\right] + \\ &\sin^2\left(\frac{1}{2}\mathbf{k}d\theta\epsilon\right)\left[\ln\left(\sin^2\left(\frac{1}{2}\mathbf{k}d\theta\epsilon\right)\right) - \ln\left(\frac{1}{2}(-\epsilon\cos(\mathbf{k}d\theta(\epsilon-1)) + (\epsilon-1)\cos(\mathbf{k}d\theta\epsilon)+1)\right)\right] \\ \approx &\frac{\mathbf{k}^2\theta^2 d^2\epsilon}{4}\,, \end{aligned} \tag{19}$$

which is optimal in the limit that $\theta, \epsilon \ll 1$.

As an example, we now show how the relative entropies are computed experimentally. For a particular value of ϵ, we measured the detection statistics for H_0 and H_1 at the value of α that maximises the output probability at one of the detectors. This value of α corresponds to the phase shift that maximises the relative entropy. For example for H_0, $\alpha = -\phi$ maximises $P_{H_0}(a)$, and the detection probabilities are

$$P_{H_0}(a) = 1/2\,(1+\nu)\,, \tag{20}$$

$$P_{H_0}(b) = 1/2\,(1-\nu)\,. \tag{21}$$

where ν is the interferometer's visibility.

For the statistics for H_0, we set $\epsilon = 0$. During the experiment, detector a registered 59461 photons, and detector b registered 176 photons.

Therefore we take

$$P_{H_0}(a) = \frac{176}{59461 + 176} = 0.99705\,,$$

$$P_{H_0}(b) = \frac{59461}{59461 + 176} = 0.00295\,.$$

For the statistics for H_1, with $\epsilon \approx 10^{-2}$, at the value of α that maximises the contrast, i.e. $\alpha \approx -(1-\epsilon)\phi - \epsilon\phi$, detector a registered 58935 photons, and detector b registered 669 photons.

Therefore our detection probabilities are

$$P_{H_1}(a) = \frac{669}{58935 + 669} = 0.9888\,,$$

$$P_{H_2}(b) = \frac{58935}{58935 + 669} = 0.0112\,.$$

We now compute the classical relative entropy

$$\mathrm{CRE} = 0.99705\,(\ln(0.99705) - \ln(0.9888)) + 0.00295\,(\ln(0.00295) - \ln(0.0112)) = 0.004369$$

which is the data point we see on the plot for $\epsilon \approx 10^{-2}$ in Fig. 5 of the main text.

V. ANGULAR SEPARATION ESTIMATION

A. The reference laser

The goal of this section is to calculate the statistics of the coherent state at the output of detectors a and b, since this is used for calibrating the adjustable phase shift α (Fig. 8).

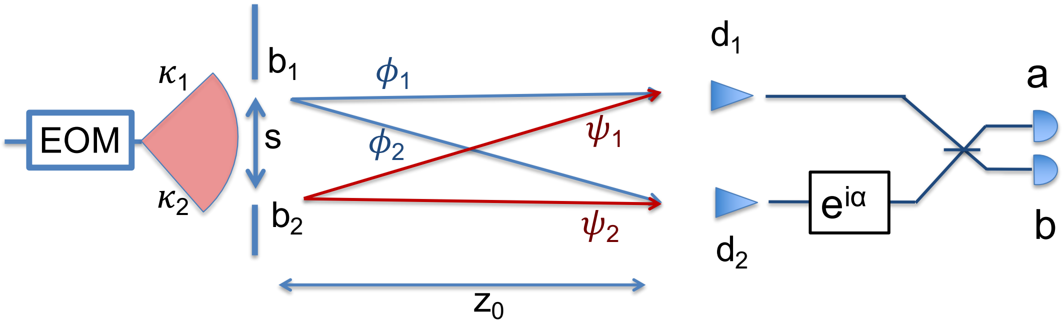

SUPPLEMENTARY FIG. 8. Schematic figure for part of the experimental set-up.

In the experiment, the laser is input into a multimode fibre. On exiting the multimode fibre, the light is highly divergent, behaving as a point source as it propagates towards the two slits. Since there is no randomness introduced in the process, we model the output at the two slits as a plane wave of coherent states. The reference laser provides a calibration for the applied phase α. The electric fields at d_1 and d_2 are given by the combination of the two fields of the two sources, whose modes we label as b_1 and b_2:

$$\widehat{E}_{d1} = \sqrt{\eta}\left(\widehat{E}_{b_1} e^{-i\phi_1} + \widehat{E}_{b_2} e^{-i\psi_1}\right), \tag{22}$$

$$\widehat{E}_{d2} = \sqrt{\eta}\left(\widehat{E}_{b_1} e^{-i\phi_2} + \widehat{E}_{b_2} e^{-i\psi_2}\right), \tag{23}$$

where η is the transmission parameter.

Since the two slits are of the same size (equal intensity), we model the coherent states at b_1 and b_2 as

$$\rho_{b1} = |\beta\rangle\langle\beta|, \qquad \rho_{b2} = \left|\beta e^{i\kappa}\right\rangle\left\langle\beta e^{-i\kappa}\right|, \tag{24}$$

where β is the amplitude of the coherent state, and κ describes a potential phase difference between the output at b_1 and b_2. The optical path differences are

$$\phi_2 - \phi_1 = \phi, \quad \psi_2 - \psi_1 = -\phi, \tag{25}$$

$$\psi_1 - \phi_1 \approx \phi, \quad \psi_2 - \phi_2 \approx -\phi, \tag{26}$$

$$\phi_1 - \psi_2 \approx 0, \quad \phi_2 - \psi_1 \approx 0, \tag{27}$$

$$\kappa = \kappa_2 - \kappa_1 . \tag{28}$$

Using the relationships in Eqs. (25)-(28) and after some simplifications, the photon numbers at d_1 and d_2 are:

$$\begin{aligned} |E_{d1}|^2 &= \eta\left(E^*_{b_1} e^{+i\phi_1} + E^*_{b_2} e^{i\psi_1}\right)\left(E_{b_1} e^{-i\phi_1} + E_{b_2} e^{-i\psi_1}\right) \\ &= \eta\left(|E_{b1}|^2 + E^*_{b1} E_{b2} e^{i(\phi_1-\psi_1)} + E^*_{b2} E_{b1} e^{i(\psi_1-\phi_1)} + |E_{b2}|^2\right) \\ &= \eta\left(|E_{b1}|^2 + |E_{b2}|^2 + 2|E_{b1}|^2 \cos(-\phi+\kappa)\right), \end{aligned} \tag{29}$$

$$\begin{aligned} |E_{d2}|^2 &= \eta\left(E^*_{b_1} e^{i\phi_2} + E^*_{b_2} e^{i\psi_2}\right)\left(E_{b_1} e^{-i\phi_2} + E_{b_2} e^{-i\psi_2}\right) \\ &= \eta\left(|E_{b_1}|^2 + |E_{b2}|^2 + E^*_{b_1} E_{b_2} e^{i(\phi_2-\psi_2)} + E^*_{b_2} E_{b1} e^{i(\psi_2-\phi_2)}\right) \\ &= \eta\left(|E_{b_1}|^2 + |E_{b2}|^2 + 2|E_{b1}|^2 \cos(\phi+\kappa)\right). \end{aligned} \tag{30}$$

We need to calculate the expectation value of this observable, $\widehat{O} = \mathrm{Re}\left[\widehat{E}^\dagger_{d1}\widehat{E}_{d2}\, e^{i\alpha}\right]$ to obtain the correlations:

$$\begin{aligned}
\widehat{O} =& \mathrm{Re}\left[\widehat{E}^\dagger_{d1}\widehat{E}_{d2}\, e^{i\alpha}\right] = \frac{1}{2}\left(E^*_{d1}E_{d2}\, e^{i\alpha} + cc.\right) \\
=& \frac{\eta}{2}\,\left[(E^*_{b1}e^{i\phi_1} + E^*_{b2}e^{i\psi_1})(E_{b_1}e^{-i\phi_2} + E_{b_2}e^{-i\psi_2})\, e^{i\alpha} + c.c.\right] \\
=& \frac{\eta}{2}\left\{e^{i\alpha}\left[|E_{b1}|^2 e^{i(\phi_1-\phi_2)} + E^*_{b1}E_{b2}e^{i(\phi_1-\psi_2)} + E^*_{b2}E_{b1}e^{i(\psi_1-\phi_2)} + |E_{b2}|^2 e^{i(\psi_1-\psi_2)}\right] + \right.\\
& \left. e^{-i\alpha}\left[|E_{b1}|^2 e^{-i(\phi_1-\phi_2)} + E_{b1}E^*_{b2}e^{-i(\phi_1-\psi_2)} + E_{b2}E^*_{b1}e^{-i(\psi_1-\phi_2)} + |E_{b2}|^2 e^{-i(\psi_1-\psi_2)}\right]\right\} \\
=& \eta\left(|E_{b1}|^2\cos(\phi_1-\phi_2+\alpha) + |E_{b2}|^2\cos(\psi_1-\psi_2+\alpha)\right) + \\
& \eta\left(E^*_{b1}E_{b2}e^{i(\phi_1-\psi_2+\alpha)} + c.c.\right) + \eta\left(E^*_{b2}E_{b1}e^{i(\psi_1-\phi_2+\alpha)} + c.c.\right) \\
=& \eta\left(|E_{b1}|^2\cos(-\phi+\alpha) + |E_{b2}|^2\cos(\phi+\alpha)\right) + \eta\left(E^*_{b1}E_{b2}e^{i\alpha} + c.c.\right) + \eta\left(E^*_{b2}E_{b1}e^{i\alpha} + c.c.\right).
\end{aligned} \tag{31}$$

The expectation value of the operator in Eq. (31) is

$$\langle\widehat{O}\rangle = \eta\Big[\beta^2\cos(-\phi+\alpha) + \beta^2\cos(-\phi+\alpha) + 4\beta^2\cos(\kappa+\alpha)\Big]. \tag{32}$$

After the collectors at d_1 and d_2, we have a phase-shifter and 50:50 beam splitter. This transformation gives

$$E_{d1} \to (A+B)\,/\,\sqrt{2}\,, \tag{33}$$

$$E_{d2} \to e^{-i\alpha}\,(A-B)\,/\,\sqrt{2}\,. \tag{34}$$

We can now calculate the statistics of the coherent state at the two detectors, A and B. We use capitalised letters to distinguish the operators from the detectors' labels a and b. Inverting the above gives

$$A = \frac{1}{\sqrt{2}}\left(E_{d1} + e^{i\alpha}E_{d_2}\right), \tag{35}$$

$$B = \frac{1}{\sqrt{2}}\left(E_{d1} - e^{i\alpha}E_{d_2}\right), \tag{36}$$

$$\begin{aligned}
A^*A =& \frac{1}{2}\Big(E^*_{d1} + e^{-i\alpha}E^*_{d2}\Big)\Big(E_{d1} + e^{i\alpha}E_{d2}\Big) \\
=& \frac{1}{2}\Big(|E_{d1}|^2 + |E_{d2}|^2 + 2\mathrm{Re}[E^*_{d1}E_{d2}e^{i\alpha}]\Big),
\end{aligned} \tag{37}$$

$$\begin{aligned}
B^*B =& \frac{1}{2}\Big(E^*_{d1} - e^{-i\alpha}E^*_{d_2}\Big)\Big(E_{d1} - e^{i\alpha}E_{d_2}\Big) \\
=& \frac{1}{2}\Big(|E_{d1}|^2 + |E_{d2}|^2 - 2\mathrm{Re}[E^*_{d1}E_{d2}e^{i\alpha}]\Big),
\end{aligned} \tag{38}$$

$$N_{\mathrm{total}} = A^*A + B^*B\,. \tag{39}$$

If the interferometer is imperfect, where some of the signal is replaced by noise, we model this as

$$A^*_{\mathrm{noisy}}A_{\mathrm{noisy}} = \nu\,A^*A + (1-\nu)\,N_{\mathrm{total}}/2\,, \tag{40}$$

$$B^*_{\mathrm{noisy}}B_{\mathrm{noisy}} = \nu\,B^*B + (1-\nu)\,N_{\mathrm{total}}/2\,. \tag{41}$$

By combining the results in Eqs. (29)-(41), we can calculate the normalised contrast between the two detectors $\mathcal{R}$.

This is defined as the difference in photon counts, divided by the total:

$$\begin{aligned}\mathcal{R} &= \frac{A^*_{\text{noisy}} A_{\text{noisy}} - B^*_{\text{noisy}} B_{\text{noisy}}}{N_{\text{total}}} \\ &= \frac{\nu \cos(\alpha) \left[\cos(\kappa) + \cos(\phi)\right]}{\cos(\kappa)\cos(\phi) + 1} \\ &= \nu \cos(\alpha) \quad \text{when} \;\; \kappa = 0\,.\end{aligned} \tag{42}$$

For $\kappa = \kappa_2 - \kappa_1 = 0$, which is our case here, since the two slits are equidistant from the output of the multimode fibre, the above expression reduces to $\cos\alpha$. That is, the reference laser behaves almost like a single point source. The parameter κ being non-zero will only reduce the visibility of the measurement, and the effect is almost negligible. We measure the parameter $\nu\cos(\alpha)$ directly from experimental data, which is then used to update the probability distribution in the maximum likelihood method.

B. Maximum likelihood

In our analysis, we use a maximum likelihood method to obtain an estimator. Using Eq. (14) in the main text, we can estimate $|\phi|$ by the estimator $\widehat{\phi}_{\text{est}}$, then obtain $\theta = s/z_0$ from

$$\widehat{\theta}_{\text{est}} = 2\widehat{\phi}_{\text{est}}/(\mathbf{k}d)\,. \tag{43}$$

We can use maximum likelihood method to obtain $|\phi|$ via Bayes' theorem. Therefore, we would like to obtain the probability distribution for $|\phi|$, $\mathcal{P}(\phi)$, given the detection events, α and $\mathcal{R}$. For two events C and D, Bayes' theorem states that

$$P(C\,|\,D) = \frac{P(D\,|\,C)\,P(C)}{P(D)}\,. \tag{44}$$

Here we use P to denote the probability density distribution, and $\mathcal{P}$ for the update distributions.

Initially the probability distribution for $|\phi|$, $P(\phi)$ is uniform in $[0, 2\pi)$, therefore $P_0(\phi) = 1/(2\pi)$. After one detection event $\mu = a, b$ and adjustable phase α, we have

$$P(\phi\,|\,\mu, \alpha, \nu) \propto \mathcal{P}(\mu\,|\,\phi, \alpha, \nu)\, P_0(\phi\,|\,\alpha, \nu)\,. \tag{45}$$

Here there is a normalisation factor that easily dealt with. We know the update distributions $\mathcal{P}(\mu\,|\,\phi)$, these are

$$\mathcal{P}(a\,|\,\phi, \alpha, \nu) = \frac{1}{2}\Big[1 + \nu\cos(\alpha)\cos(\phi)\Big]\,, \tag{46}$$

$$\mathcal{P}(b\,|\,\phi, \alpha, \nu) = \frac{1}{2}\Big[1 - \nu\cos(\alpha)\cos(\phi)\Big]\,. \tag{47}$$

Given a detection event $\mu = a, b$ where the adjusted phase was α, the probability for ϕ can be updated via

$$P(\phi\,|\,\mu, \alpha, \nu) \propto \mathcal{P}(\mu\,|\,\phi, \alpha, \nu)\, P_0\,. \tag{48}$$

Therefore, after the detection event, Eq. (48) is updated using Eqs. (46)-(47), depending on whether a or b occurred. After m detections, we have the vector of detection events, $\vec{\mu}_m = (a, b, b, a, ...)$, for a given vector of adjustable phases $\vec{\alpha} = (\alpha_1, \alpha_2, ..., \alpha_m)$. In the experiment, α is constant for each data point. We have

$$P(\phi\,|\,\vec{\mu}_m, \vec{\alpha}, \nu) \propto \mathcal{P}(\mu\,|\,\phi, \alpha, \nu)\, P(\vec{\mu}_{m-1}\,|\,\phi, \vec{\alpha}, \nu)\,. \tag{49}$$

Now, since all the functions we deal with here are sinusoids, we can conveniently express them as a Fourier series.

After m clicks, the probabilities can be expressed as a Fourier series

$$P(\phi\,|\,\vec{\mu}_m, \vec{\alpha}) = \frac{1}{2\pi}\sum_{k=-m}^{m} a_k e^{i\mathbf{k}\phi}\,, \tag{50}$$

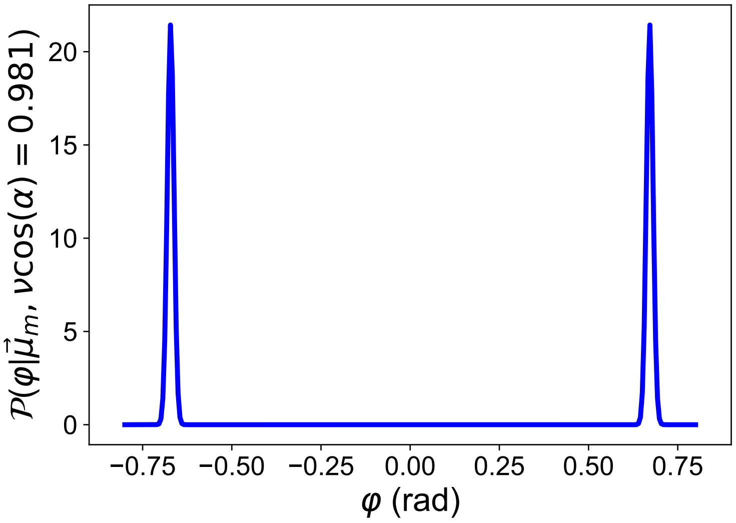

SUPPLEMENTARY FIG. 9. Probability density function for ϕ after 12740 detection events of which 1478 were registered by detector b.

where m here corresponds to the higher order of the Fourier coefficient. The coefficient of the term $e^{i\mathbf{k}\phi}$ is denoted a_k, which depends on $\vec{\mu}_m$ and α_m. Normalising α_0 to $1/2\pi$ will keep the entire distribution normalised.

We equivalently write the update events in this Fourier form. For example, if detector b fires, then

$$\mathcal{P}(\mu = b \,|\, \phi, \alpha, \nu) = \frac{1}{2}\Big[1 - \nu\cos(\alpha)\cos(\phi)\Big]\,. \tag{51}$$

We write Eq. (51) as

$$\mathcal{P}(\mu = b \,|\, \phi, \alpha, \nu) = \frac{1}{2} - \frac{1}{4}\nu\cos(\alpha)\,e^{i\phi} - \frac{1}{4}\nu\cos(\alpha)\,e^{-i\phi}\,, \tag{52}$$

therefore, the update coefficients are $a_0 = \pi, a_1 = a_{-1} = \frac{\pi}{2}\nu\cos(\alpha)$. This example is particularly relevant, because the factor $\nu\cos(\alpha)$ is equal to $\mathcal{R}$ in Eq. (42), and is directly measured in the experiment using the calibration laser. Before the first detection, Eq. (50) only contains one term, $a_0 = 1$. After each detection event given by the probabilities in Eqs. (46)-(47), the number of Fourier coefficients grows by 2; a_k are updated using Eq. (49), which once again uses Eqs. (46)-(47). After the coefficients are obtained, $P(\phi)$ can be evaluated for each value of ϕ, and we extract the maximum. There are two peaks for ϕ of equal intensity, since $\cos(\phi) = \cos(-\phi)$. We show an example in Fig. 9 with $\nu\cos\alpha = 0.981$ after 12740 detection events where 1478 were output at detector b.

VI. PRACTICAL EXAMPLE WITH AN IMAGING INSTRUMENT

As an example, the exoplanet LkCa 15 c [2] was observed with a Large Binocular Telescope (LBT) with variable baselines ranging from 1.4 to 7.0 m, at wavelengths 2.18 μm and 3.8 μm.

In imaging mode, the PSF of a circular lens is the Airy function, which we approximate with a Gaussian:

$$\psi(x) = \left(\frac{1}{2\pi\sigma^2}\right)^2 e^{-x^2/4\sigma^2} \tag{53}$$

for which the standard deviation is well-approximated by

$$\sigma \approx \frac{\sqrt{2}}{\mathbf{k}\, r} \tag{54}$$

where $\mathbf{k}$ is the wavenumber and r is the diameter of the combined mirrors.

If the instrument is diffraction-limited, then the smallest PSF obtainable has $\sigma \approx 29$ milliarcseconds (with a wavelength of 2.18 μm and a 7 m baseline). In Fig. 10 we show the precision limits of such an instrument operating in direct imaging (DI) mode, as well as using our method.

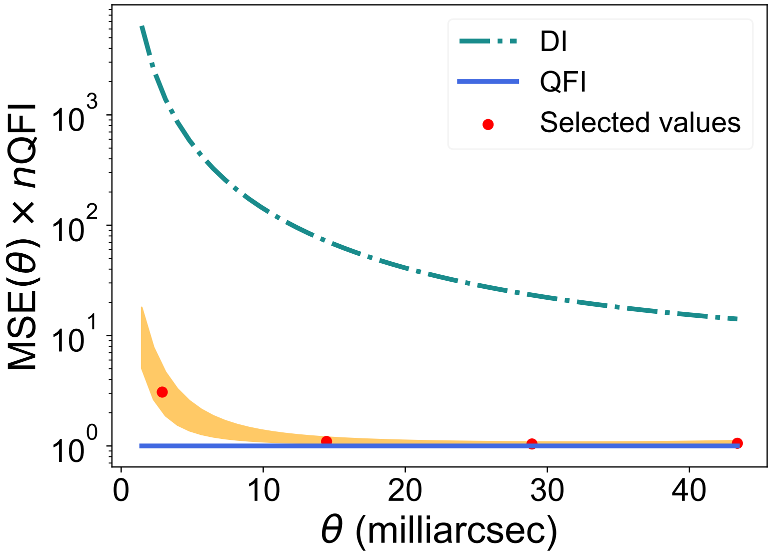

SUPPLEMENTARY FIG. 10. Precision limits for the Large Binocular Telescope (LBT).

The orange shaded region denotes the achievable precision if the visibility is between 96% and 98%. The red points on the plot correspond to the selected values of angular separation $\theta = 0.1\sigma$, 0.5σ, 1σ and 1.5σ respectively, with a visibility parameter $\nu = 0.98$ (for comparison, the largest optical interferometer, CHARA, has a visibility value $\nu > 99\%$). As we see, using our method, an instrument with a PSF of ≈ 30 milliarcseconds can resolve two binary stars with separations $\theta < 10$ milliarcseconds with reasonable accuracy.

VII. NON-ZERO AXIAL SEPARATION

We now investigate the effect of having non-zero axial separation of the two sources on hypothesis testing.

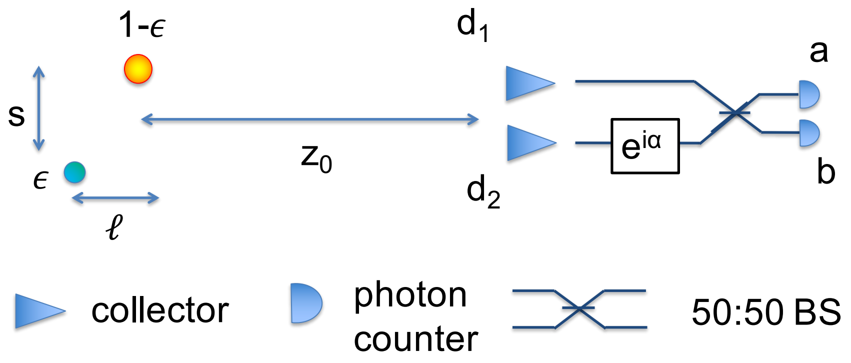

SUPPLEMENTARY FIG. 11. Schematic with non-zero axial separation.

For the scheme shown in Fig. 11, the wavefunction of the star is:

$$|\psi_{\rm star}\rangle = \frac{1}{\sqrt{2}}\Big(\,|d_1\rangle + e^{i\phi}\,|d_2\rangle\Big)\,, \tag{55}$$

$$\phi = \mathbf{k}\,d\theta/2\,, \quad \theta = s/z_0\,. \tag{56}$$

where the relative phase of a photon coming from the star is the same as the one described in the sections above.
For a photon coming from the planet, we have instead:

$$|\psi_{\rm planet}\rangle = \frac{1}{\sqrt{2}}\Big(\,|d_1\rangle + e^{i\psi_p}\,|d_2\rangle\Big)\,. \tag{57}$$

$$\begin{aligned}\psi_p &= \mathbf{k}\left(\sqrt{(z_0+\ell)^2+\left(-\frac{s}{2}+\frac{d}{2}\right)^2}-\sqrt{(z_0+\ell)^2+\left(-\frac{s}{2}-\frac{d}{2}\right)^2}\right)\,,\\ &\approx \mathbf{k}(z_0+\ell)\left[1+\frac{1}{2(z_0+\ell)^2}\left(\frac{s}{2}-\frac{d}{2}\right)^2\right]-\mathbf{k}\,(z_0+\ell)\left[1+\frac{1}{2(z_0+\ell)^2}\left(\frac{s}{2}+\frac{d}{2}\right)^2\right]\\ &= -\frac{d\,s}{\ell+z_0}\\ &\approx -\frac{\mathbf{k}d\theta}{2}+\frac{\mathbf{k}d\theta}{2}\frac{\ell}{z_0}\equiv -\frac{\mathbf{k}d\theta}{2}+\frac{\mathbf{k}d\theta}{2}\xi\end{aligned} \tag{58}$$

where we have defined $\xi = \ell/z_0$. For convenience, here we assume that the centroid of the star-planet system is aligned with the centre of the interferometer, however this assumption is not necessary.
In the limit that $\epsilon, \theta, \xi \ll 1$, the QRE of the two hypotheses is

$$D(\rho_0||\rho_1) \approx \frac{1}{4}d^2\theta^2\mathbf{k}^2\epsilon(1-\xi) \tag{59}$$

The effect of having non-zero axial separation of the two sources is to increase (or reduce) the QRE by a factor of $(1 \pm \xi)$, which is small since $\ell \ll z_0$.

VIII. THE QUANTUM HOEFFDING BOUND

Define the asymptotic error exponents for the type-I and type-II errors:

$$\alpha_R = -\lim_{n\to\infty} \frac{1}{n} \ln \alpha_n \,, \tag{60}$$

$$\beta_R = -\lim_{n\to\infty} \frac{1}{n} \ln \beta_n \,. \tag{61}$$

The Quantum Hoeffding bound gives the best exponential convergence rate of the type-I error α_R, under the constraint that the rate limit of the type-II error $\beta_R \geq r$, then we can achieve type-I error rates $\alpha_R = e_Q(r)$ [3].

Let supp ρ_0 and supp ρ_1 be the support projections associated with states ρ_0 and ρ_1 respectively. Define

$$\Gamma_0 = \mathrm{Tr}[\rho_0 \,\mathrm{supp}\,\rho_1] \,, \qquad \Gamma_1 = \mathrm{Tr}[\rho_1 \,\mathrm{supp}\,\rho_0] \,, \tag{62}$$

then

$$e_Q(r) = \begin{cases} \max\limits_{(0\,\leq\, s\,<\,1)} \dfrac{-sr - \ln\big(\mathrm{Tr}[\rho_0^{1-s}\rho_1^s]\big)}{1-s} & \text{for } \; -\ln(\Gamma_1) \leq r \leq D(\rho_0||\rho_1) \\ \\ \infty & \text{for } \; 0 \leq r < -\ln(\Gamma_1) \end{cases} \tag{63}$$

The two density matrices are

$$\rho_0 = \begin{pmatrix} 1 & 0 \\ 0 & 0 \end{pmatrix} \,, \tag{64}$$

$$\rho_1 = \begin{pmatrix} 1-\epsilon\sin^2(\phi) & -i\epsilon\sin(\phi)\cos(\phi) \\ i\epsilon\sin(\phi)\cos(\phi) & \epsilon\sin^2(\phi) \end{pmatrix} \quad \text{with } \; \phi = \mathbf{k}d\theta/2 \,. \tag{65}$$

Therefore in our case

$$\Gamma_1 = 1 - \epsilon\sin^2(\phi) \,, \tag{66}$$

$$-\ln(\Gamma_1) \approx \epsilon\sin^2(\phi) \,. \tag{67}$$

The expression for the QRE is:

$$D(\rho_0||\rho_1) = \frac{\tanh^{-1}\left(\sqrt{\Delta}\right)\left[\epsilon\,(1-\cos(2\phi)) - 1\right]}{\sqrt{\Delta}} - \frac{1}{2}\ln\left[\frac{1-\Delta}{4}\right] \,, \tag{68}$$

$$\begin{aligned} \Delta =& -2(\epsilon-1)\epsilon\cos(2\phi) + 2(\epsilon-1)\epsilon + 1 \\ \approx& \;\epsilon\sin^2(\phi) \,. \end{aligned} \tag{69}$$

From this we see that $D(\rho_0||\rho_1) = -\ln(\Gamma_1)$ if ϵ is sufficiently small. This means that α_R is almost always infinite, which is what we would have expected. This can be explained by the fact that, if we perform the perfect measurement, a false positive can never occur. Light coming from a single star will couple into the same mode, and no light will be detected in the other modes in the absence of the planet. Intuitively, this can also be seen from the fact that, when we calculate the relative entropy, we have $D(\rho_0||\rho_1) = \infty$.

SUPPLEMENTARY REFERENCES

[1] C. Lupo, Z. Huang, and P. Kok, Quantum limits to incoherent imaging are achieved by linear interferometry, Phys. Rev. Lett. **124**, 080503 (2020).

[2] S. Sallum, K. Follette, J. A. Eisner, L. M. Close, P. Hinz, K. Kratter, J. Males, A. Skemer, B. Macintosh, P. Tuthill, *et al.*, Accreting protoplanets in the lkca 15 transition disk, Nature **527**, 342 (2015).

[3] K. M. Audenaert, M. Nussbaum, A. Szkoła, and F. Verstraete, Asymptotic error rates in quantum hypothesis testing, Communications in Mathematical Physics **279**, 251 (2008).
